# Comparative Analysis of the Effectiveness of Four Distinct Remineralizing Agents in Artificial White Spot Lesions Following Chitosan Nanoparticle Pretreatment: An In Vitro Study

**DOI:** 10.7759/cureus.59924

**Published:** 2024-05-08

**Authors:** G Nandhini, Rajesh Sasidharan Nair, Jeya Balaji Mano Christiane Angelo, Rayar Sreeram, Nyklesh V, VS Swathi

**Affiliations:** 1 Conservative Dentistry and Endodontics, Chettinad Dental College & Research Institute, Chennai, IND; 2 Conservative Dentistry and Endodontics, Sree Mookambika Institute of Dental Sciences, Kulasekharam, IND

**Keywords:** remineralization, demineralization, dental caries, pretreatment, chitosan nanoparticles

## Abstract

Aim

The aim of the study was to compare the effectiveness of chitosan nanoparticle pretreatment with four different remineralizing agents in artificial white spot lesions.

Methods

A total of 100 human maxillary first premolars were selected and divided into five groups of 20 samples in each group. Artificial white spot lesions were created by immersing the samples in the demineralizing solution for 96 hours. Chitosan pretreatment was done for all samples followed by subjecting Group I samples to artificial saliva (control), Group II samples to 3M Clinpro, Group III samples to GC Tooth Mousse, Group IV samples to SHY-NM, and Group V samples with Aclaim using a cotton applicator tip. Each group was divided into two subgroups of 10 samples, which were subjected to hardness testing and mineral content analysis. Surface microhardness and the calcium phosphorous ratio were recorded using a Vickers microhardness tester and energy-dispersive X-ray (EDAX) analysis at three levels i.e., baseline, after demineralization, and after remineralization and tabulated. Statistical analysis was conducted by analyzing data using ANOVA and post hoc followed by Dunnett's t-test using IBM SPSS Statistics for Windows, Version 16 (Released 2007; IBM Corp., Armonk, New York, United States).

Results

Vickers surface hardness testing and EDAX analysis showed statistically significant values for all the groups. Among them, maximum remineralization potential was seen in samples treated with Chitosan and 3M Clinpro combination, and minimum remineralization potential was seen in Chitosan and artificial saliva combination.

Conclusion

The addition of chitosan nanoparticles with various remineralizing agents showed a significant synergistic effect on remineralization activity. Also, chitosan and Clinpro combination showed the maximum surface hardness and EDAX analysis values when compared to other groups.

## Introduction

Dental caries is one of the most common dental diseases and is recognized as a leading cause of tooth loss [1]. Fluoride has previously been used for caries treatment due to its cariostatic potential [2]. However, certain limitations (such as dental and skeletal fluorosis) have restricted its use. Research is being done to develop recent preventive agents that act as adjuncts to fluoride [1].

Clinpro 5000 (3M ESPE, Canada) contains 1.1% NaF silica and a functionalized tricalcium 6 phosphate (fTCP) known as hydroxyapatite [2]. This has been shown to boost remineralization performance relative to fluoride-only systems. It controls calcium and phosphate delivery and prevents early interactions with fluoride [2]. CPP-ACP (GC Tooth Mousse) is developed from milk protein and acts as a reservoir of calcium and phosphate ions, resulting in the formation of hydroxyapatite or fluorapatite crystals, thereby promoting remineralization [3]. Novamine (SHY NM, Group Pharmaceuticals, India) is a bioactive glass that was originally used as a bone regenerative material [4]. It forms a hydroxycarbonate apatite (HCA) layer similar to naturally occurring biological apatite, thereby halting the demineralization and enhancing the remineralization process [1,4]. Nanohydroxyapatite (Aclaim, Group Pharmaceuticals, India) toothpaste is morphologically similar to the apatite crystal of tooth enamel. It fills defects on demineralized teeth, continuously attracts calcium and phosphate ions to the defective surface, and promotes crystal integrity and growth [5].

Chitosan (Sigma Aldrich, Bangalore) is obtained from arthropod shells. It electrostatically attracts negatively charged microbial species, inhibiting their growth and the formation of dental plaque. Due to continuous adhesion provided by chitosan, biomineralization did not ease, even when the pH decreased below 6.5 [6].

The Vickers hardness test measures the indentation hardness of thin sections of metals, ceramics, and other materials [7]. Energy-dispersive X-ray (EDAX) image analysis is the gold standard for mineral content estimation at the ultrastructural level. It is critical to select an appropriate material with high remineralizing potential, so this study compared the effectiveness of chitosan nanoparticle pretreatment with four remineralizing agents in artificial white spot lesions.

## Materials and methods

A total of 100 human maxillary first premolars freshly extracted for orthodontic treatment with no caries were included. Exclusion criteria were teeth examined with extensive caries, restorations, enamel hypoplasia, and cracks. Each tooth was thoroughly cleaned and stored in a 10% formalin solution. The sample size was calculated using the following formula.

n= 2S^2^(z_1_+z_2_)^2^/(m_1_-m_2_)^2^

The teeth were decoronated 1 mm below the cementoenamel junction using a slow-speed diamond disc. The decoronated samples were stored in a 0.1% thymol solution until the study was initiated. Custom plastic cylindrical molds were prepared and filled with chemical-cured resin. Each sample was placed in the resin mold with the buccal surface facing upward, parallel to the horizontal plane.

In the middle of the sample, a 5 mm × 5 mm enamel window was created using adhesive tape. The window was made acid-resistant by applying nail varnish around it. After the samples were dried, the adhesive tape was removed using an explorer, exhibiting a rectangular area on the enamel surface.

The surface microhardness and calcium phosphorous ratio were recorded using a Vickers microhardness tester and EDAX analysis, respectively, at three levels: baseline, after demineralization, and after remineralization. All the samples were divided into five groups (I, II, III, IV, and V) of 20 samples, which were further divided into two subgroups (a and b) of 10 samples. All the samples in subgroup a were subjected to Vickers hardness testing, and the samples in subgroup b were subjected to EDAX analysis.

The demineralizing solution was prepared using 2.2 mM potassium phosphate, 2.2 mM calcium chloride, and 0.05 M acetic acid, and the pH was maintained at 4.4 with 1 M sodium hydroxide. The remineralizing solution was prepared using 0.9 mM sodium phosphate, 1.5 mM calcium chloride, and 0.15 M potassium chloride, and the pH was maintained at 7.0. Both demin/remin solutions were prepared in the department of biochemistry at Sree Mookambika Institute of Dental Sciences, Kulasekharam. All the samples were individually immersed in the demineralizing solution (20 ml) for 96 hours to produce artificial carious lesions in the enamel. A Vickers surface hardness test and an EDAX assessment were done for all the samples in each subgroup to record the values of artificially produced demineralized lesions.

Then, 20 μl of chitosan (Sigma Aldrich, Bangalore) nanoparticle solution was applied on dried enamel surfaces using a cotton applicator twice daily for one minute. All the chitosan pretreated samples were subjected to four different remineralizing agents as mentioned in Table 1.

**Table 1:** Treatment of samples with remineralizing agents

Groups	Remineralizing Agents
Group I	Artificial saliva (Apexion, Kozhikode)
Group II	Clinpro 5000 dentifrice (3M ESPE, Canada)
Group III	GC Tooth Mousse dentifrice (Group Pharmaceuticals, India)
Group IV	SHY NM dentifrice (Group Pharmaceuticals, India)
Group V	Aclaim dentifrice (Group Pharmaceuticals, India)

A pH cycling model was created to mimic the changes in the oral environment. The remineralizing pastes were applied using applicator tips for two minutes, and samples were washed with deionized water. The samples were then individually immersed in 20 ml of demineralizing solution (pH 4.4) for three hours and washed with deionized water. Then, the samples were treated again with the respective remineralizing agents for two minutes, which were then washed off with deionized water. All the enamel samples were then individually immersed in 20 ml of remineralizing solution (pH 7) for 17 hours. The pH cycling was carried out for 30 days. The remineralizing and demineralizing solutions were replaced every 48 hours and 5 days, respectively.

All samples in each subgroup were individually subjected to Vickers microhardness and EDAX surface analyses to record the values of remineralization. The values were obtained at each of the three stages of the study: baseline, after demineralization, and after remineralization.

## Results

Data were collected and statistical analysis was done using one-way ANOVA (post hoc) followed by Dunnett's t-test and tabulated. Table 2 denotes the mean load values of Vickers Hardness of different groups at baseline, after demineralization, and after one month of remineralization.

**Table 2:** Vickers Hardness of different groups at baseline, after demineralization, and after one month of remineralization

Groups	Baseline (Mean±SD)	Demineralization (Mean±SD)	After one month (Mean±SD)
Group I	239.98±69.43	196.31±41.38	205.42±39.84
Group II	251.59±35.24	235.60±15.63	246.29±10.78
Group III	259.43±31.05	219.39±15.98	225.08±12.42
Group IV	248.91±18.34	209.25±46.19	216.73±44.35
Group V	240.53±49.72	228.64±29.19	233.61±18.63

Table 3 denotes multiple comparisons of mean load values at baseline between the groups.

**Table 3:** Intergroup comparison of baseline values

Groups	Baseline (Mean±SD)	Comparison	p-value
Group I	239.98±69.43	I with II, III, IV, V	0.78
Group II	251.59±35.24	II with I, II, III, IV, V	0.31
Group III	259.43±31.05	III with I, II, IV, V	0.42
Group IV	248.91±18.34	IV with I, II, III, V	0.89
Group V	240.53±49.72	V with I, II, III, IV	0.45

Table 4 denotes multiple comparisons of mean load values after demineralization between the groups.

**Table 4:** Intergroup comparison of demineralization values

Groups	Demineralization (Mean±SD)	Comparison	p-value
Group I	196.31±41.38	I with II, III, IV, V	0.02
Group II	235.60±15.63	II with I, II, III, IV, V	0.03
Group III	219.39±15.98	III with I, II, IV, V	0.04
Group IV	209.25±46.19	IV with I, II, III, V	0.02
Group V	228.64±29.19	V with I, II, III, IV	0.04

Table 5 denotes multiple comparisons of mean load values after one month of remineralization between the groups.

**Table 5:** Intergroup comparison of remineralization values

Groups	After one month (Mean±SD)	Comparison	p-value
Group I	205.42±39.84	I with II, III, IV, V	0.03
Group II	246.29±10.78	II with I, II, III, IV, V	0.04
Group III	225.08±12.42	III with I, II, IV, V	0.03
Group IV	216.73±44.35	IV with I, II, III, V	0.04
Group V	233.61±18.63	V with I, II, III, IV	0.04

Table 6 denotes mean load values of the calcium phosphorus ratio of different groups at baseline, after demineralization, and after one month of remineralization.

**Table 6:** Intergroup comparison of calcium phosphorus ratio at baseline, after demineralization, and after remineralization Results obtained were statistically significant with p<0.05

Groups	Baseline (Mean±SD)	After demineralization (Mean±SD)	After remineralization (Mean±SD)
Group I	2.73±0.15	2.18±0.06	2.30±0.15
Group II	2.60±0.14	2.22±0.16	2.56±0.15
Group III	2.95±0.14	2.04±0.15	2.44±0.15
Group IV	2.93±0.15	2.83±0.14	2.41±0.14
Group V	2.69±0.17	2.25±0.15	2.51±0.14

On comparing the values obtained, it was found that demineralized values were found to be lower than the baseline values in all the groups. Also, remineralized values were found to be higher than the demineralized values but always lower than the baseline values in all the groups.

According to Vickers testing, Group II showed the maximum surface microhardness after one month of remineralization treatment followed by Group V, Group III, and Group IV and the least values were seen for Group I. According to EDAX analysis, Group II showed the maximum calcium phosphorous ratio after one month of remineralization treatment followed by Group V, Group III, and Group IV, and the least values were seen for Group I. The results obtained were statistically significant for all the groups indicating that the various agents used showed good remineralization properties and also inhibited caries formation. 

## Discussion

Dental caries is a prevalent noncommunicable oral disease. Clinically, it manifests as a white spot lesion in the early stage, which can be observed with the naked eye as a white, chalky lesion. Demineralization results from predominant diet variations, poor oral hygiene, or increased microbial activity. Saliva typically increases the buffering capacity, promoting remineralization [1].

Recently, the concept of a non-invasive approach has become popular. This approach includes detecting and treating the carious areas sooner, relying more on prevention than the traditional method [1]. Various remineralizing agents promote healing by replacing the minerals lost during demineralization [1-4]. Fluoride was recognized as the main remineralizing agent due to its cariostatic potential. Apart from halting caries progression, it has some limitations, such as skeletal and dental fluorosis [4-6]. Because of these limitations, other non-fluoride agents, such as CPP-ACP, Novamine, self-assembling peptides, and nanohydroxyapatite, were introduced as adjuncts to fluorides.

In this study, all samples were pretreated with chitosan nanoparticles for one minute, in accordance with a study done by Zhang et al. [6], to avoid deep erosion or etching of the tooth surface. This was followed by treatments with different remineralizing agents for two minutes according to the ADA’s brushing time recommendations to obtain effective quantitative and therapeutic values. Chitosan (Sigma Aldrich, Bangalore) is a mucopolysaccharide present in crustaceans’ exoskeletons. It showed an inhibitory effect against streptococcus and enterococcus species with its electrostatic interaction. It functions as a bio-adhesive polymer with a prolonged retention time on the oral mucosa and synergistic antiplaque, antibacterial effect with chlorhexidine and other remineralizing agents [6,8]. According to an in vitro study conducted by Ryge et al. [7], enamel treated with acid-soluble chitosan showed a higher Vickers surface microhardness than untreated samples. Nanoparticles range in size from 1 to 100 nm (1 nm = 10−9 m). Chitosan nanoparticles increase the surface area up to hundreds of times compared to microparticles (10−6 m) and increase the ability to bind to chemical groups promoting reactivity.

Fluoride effectively increases the remineralization of early enamel lesions [1]. It is adsorbed into the enamel surface, resisting acid attacks, depositing minerals resulting in the formation of acid-resistant fluorapatite crystals, and acting as a bioreservoir of Ca, P, and F ions on adjacent porous enamel [9-12]. In this study, Clinpro (3M ESPE, Canada) dentifrice had high remineralization potential compared to other groups, which was in accordance with in-vitro studies conducted by Baysan et al. Clinpro contains sodium fluoride with functionalized tricalcium phosphate (f-TCP) as the main ingredient, which acts as a continuous reservoir of calcium and phosphate ions, providing optimal benefits even when delivered in a neutral pH environment. An important feature of this calcium phosphate system is that it is stable in an aqueous environment and does not affect the fluoride activity added to the dentifrice. f-TCP is a smart ingredient that controls calcium and phosphate delivery by surrounding them with a protective coating, preventing them from early interactions with fluoride until brushed on the tooth [13,14].

Nanohydroxyapatite dentifrice is a biomimetic remineralizing paste that contains calcium and phosphate in the hydroxyapatite form as a key ingredient [1]. The small HAP nanoparticles resemble the features of biological apatite and self-assemble to form enamel-like structures [15], filling the fine pores to form a homogeneous apatite layer [16] and maintaining a high concentration of calcium and phosphate ions in the subsurface enamel. In this study, Aclaim (Group Pharmaceuticals, India) dentifrice was used, which showed a better remineralizing effect and synergistic effect with chitosan, in accordance with other studies. Though it possessed improved remineralizing potential, it was found to be less than fluoride dentifrice. In accordance with the study done by Kim et al., nano-HAP-based dentifrice had comparable remineralizing potential to fluoride dentifrice and can be used as adjuvant to fluoride therapy [15].

Casein phosphopeptide (CPP) consists of multiphosphoseryl clusters that stabilize calcium phosphate by forming colloidal casein-phosphopeptide amorphous calcium phosphate (ACP) nanocomplexes, preventing the destruction of calcium and phosphate ions [1]. CPP-ACP nanocomplex can maintain high-concentration gradients of calcium and phosphate ions within the subsurface lesion, resulting in the formation of hydroxyapatite or fluorapatite crystals via crystal growth [17-20]. In this study, GC Tooth Mousse (Group Pharmaceuticals, India) showed superior hardness to Novamine technology but inferior to fluoride and nanohydroxyapatite toothpaste. The peculiar nature of CPP maintains a supersaturated state of calcium and phosphate ions through the reservoir of bound ACP that promotes diffusion into the lesion, thereby promoting remineralization [20,21].

Novamine is a bioactive glass widely used in the fields of tissue engineering, bone regeneration, and dentin remineralization; it is also used as a desensitizing agent [22-24]. In this study, SHY-NM (Group Pharmaceuticals, India) contains a calcium sodium phosphosilicate that reacts with saliva, allowing sodium ions to exchange with hydrogen ions, thereby raising the pH to form a new acid-resistant HCA layer over demineralized areas, promoting remineralization [1,24]. Chitosan pretreatment also improved remineralization when used with bioactive glass slurry through electrostatic interactions [8,25]. In this study, a statistically significant decrease in calcium phosphorous ratio and microhardness values was found. In all groups, the samples had plugs that sealed demineralized pores and were capable of remineralization, but the bioactive glass had more potential to remineralize than CPP-ACP in a few studies. The clinical advantage is its cost-effectiveness and availability in the form of dentifrice, which may increase patient compliance [24,25]. As in vitro values are variable, further research should be done to evaluate particular remineralizing agents in resisting demineralization and promoting remineralization.

## Conclusions

All the remineralizing agents used in this study showed significant results in preventing ion loss and promoting remineralization. Vickers and EDAX analysis were highest for chitosan and Clinpro combination followed by Aclaim, GC Tooth Mousse, and SHY NM. Also, remineralized values were more than demineralized but always less than baseline values indicating that non-carious, non-hypoplastic enamel is strongly resistant to any mineral ions.

Hence, we can conclude that chitosan can be used in optimal concentration on a daily basis as mouthwash followed by regular brushing with any of these remineralizing dentifrices to prevent caries formation at an early stage. 
